# Attaining Automaticity in the Visual Numerosity Task is Not Automatic

**DOI:** 10.3389/fpsyg.2015.01744

**Published:** 2015-11-17

**Authors:** Craig P. Speelman, Katrina L. Muller Townsend

**Affiliations:** School of Psychology and Social Science, Edith Cowan University, JoondalupWA, Australia

**Keywords:** automaticity, skill acquisition, average, individual differences, practice

## Abstract

This experiment is a replication of experiments reported by Lassaline and Logan (1993) using the visual numerosity task. The aim was to replicate the transition from controlled to automatic processing reported by Lassaline and Logan (1993), and to examine the extent to which this result, reported with average group results, can be observed in the results of individuals within a group. The group results in this experiment did replicate those reported by Lassaline and Logan (1993); however, one half of the sample did not attain automaticity with the task, and one-third did not exhibit a transition from controlled to automatic processing. These results raise questions about the pervasiveness of automaticity, and the interpretation of group means when examining cognitive processes.

## Introduction

Speelman and McGann’s (2013) paper contained a clear message for Psychology: Be wary of phenomena that are discovered on the basis of average group data. Speelman and McGann (2013) argued that the averaging process typically used in the analysis methods adopted by Psychology can mask individual results that may actually be counter to those revealed by the group results. As a result, group results may not be an accurate reflection of the behavior of many, and possibly most, individuals in the group.

Most of the phenomena we teach as the basic facts in an introductory course in Cognitive Psychology have typically been generated by experiments where groups of subjects perform the same task under various conditions. The classic result is usually observed and interpreted as a pattern of differences between group and/or condition means. That is, the take-home message from these experiments is usually represented as a pattern of results that are generated by averaging across the results of individuals. This results in a ‘clean’ picture of behavior where the noise associated with individual differences has effectively been removed. Thus well-known effects such as the word superiority effect, the serial position curve, the power law of learning, and the phonological similarity effect have been well replicated by different researchers and under different conditions, but they all are observed by averaging data collected from groups of individuals. Speelman and McGann (2013) argued that such effects may not be as pervasive as their replicability suggests. That is, although the effects can be replicated easily enough, they may only exist when the data from several individuals are combined, and as a result may not reflect the cognitive processes of many, and at worst, any individuals in that group.

Speelman and McGann (2013) reported results from a replication of the Word Superiority Effect. Although the average performance of the sample in their experiment replicated the classic effect, an examination of the performance of individuals in the sample revealed that very few people produced results consistent with the effect. Speelman and McGann (2013) argued that such a result should reduce the confidence we have in using means to reveal information about fundamental cognitive processes. A further implication of this result is that it may be prudent to determine the extent to which individuals demonstrate performance patterns that have to date been demonstrated in group results.

The research reported in this paper was designed to examine a well-replicated finding in the field of attention. From at least as far back as the 1970s, researchers (e.g., Posner and Snyder, 1975; Schneider and Shiffrin, 1977; Hasher and Zacks, 1979) have drawn a distinction between automatic and controlled/conscious/effortful forms of mental processing. Controlled processes are typically exhibited early in the practice of a task, while we are more likely to perform automatically after a long period of practice. Controlled, deliberate psychological processes are used for difficult and unfamiliar tasks. These processes operate serially, use substantial cognitive resources, require attention, and are flexible. In contrast, automatic processes are used for easy and familiar tasks, operate in parallel, require very few cognitive resources, do not require attention, and are difficult to modify (Speelman and Maybery, 1998). Automatic performance only comes after extensive practice. Thus, with sufficient practice under appropriate conditions (Schneider and Shiffrin, 1977; Shiffrin and Schneider, 1977) one can develop the ability to respond in an automatic fashion to particular stimuli.

Most theories of cognitive skill acquisition describe mechanisms by which practice produces a shift from controlled to automatic processing (e.g., Logan, 1988; Anderson and Lebiere, 1998). Although these theories propose different means by which practice leads to more efficient processing, all of the theories lead to the same prediction: with sufficient practice of a task where the stimulus–response relationship is consistent, performance will reach the stage where perception of a known stimulus will trigger an automatic response (i.e., seeing ‘3 × 4 = ?’ will automatically lead to a response of ‘12’).

This view of the development of automatic processing has influenced ideas of how we acquire complex skills. When we initially embark on the acquisition of such skills, effort, and attention are focused on basic, low level tasks (e.g., recognizing letters when learning to read). These tasks are practiced until processes are developed that perform this task automatically. Initially, these processes require most of the available cognitive resources to proceed. Little capacity is available for any other task (e.g., reading words). Once these processes have become automatic, however, sufficient cognitive resources are available for the person to attempt higher level tasks (e.g., reading words). Importantly, higher level tasks (i.e., reading words) are considered to operate on the outcomes from lower level tasks (e.g., letter identification; Karmiloff-Smith, 1979). With further practice, processes will be developed that are specific to the higher level task and these in turn may become automatic, enabling further developments in the level of skill (Speelman and Kirsner, 2005). This view is clearly articulated in mainstream educational practice (e.g., Cumming and Elkins, 1999; Caron, 2007; Baroody et al., 2009).

**FIGURE 1:**
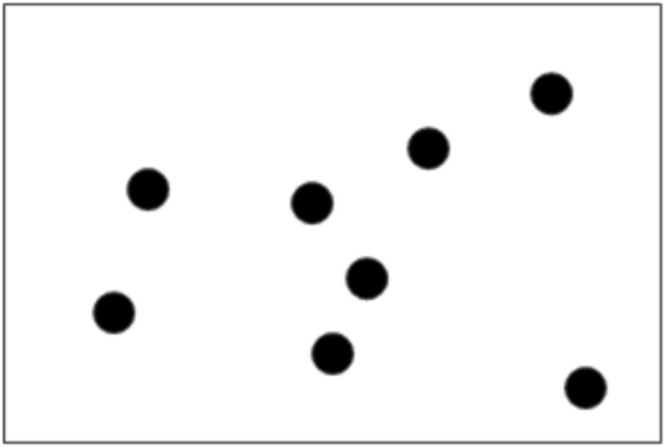
**An example of the type of dot picture used by Lassaline and Logan (1993).** Subjects are asked to indicate the number of dots in the picture.

**FIGURE 2:**
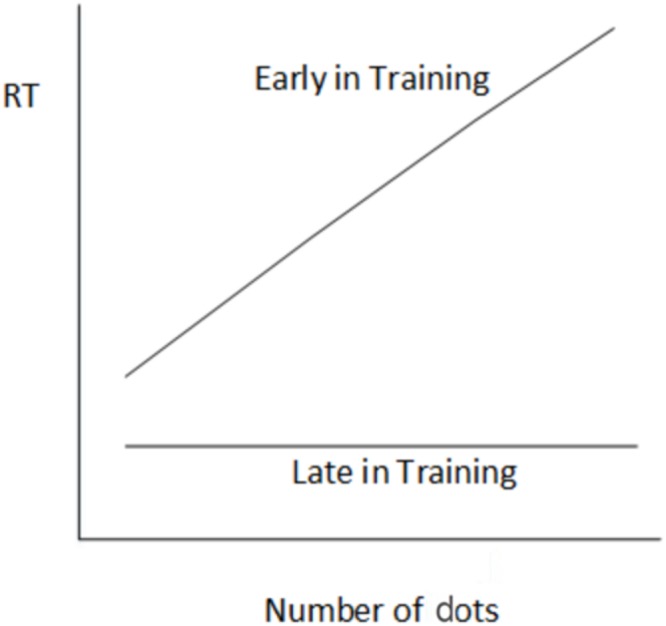
**Reaction time (RT) in the visual numerosity task as a function of number of dots in each stimulus picture**.

Automaticity can be attained quickly with simple tasks. Lassaline and Logan (1993) trained subjects on a visual numerosity task. In this task pictures of dots or similar, ranging in number from 6 to 11 and arranged in a seemingly random manner, are presented on a computer screen, one at a time (**Figure 1**). Subjects are required to indicate how many dots are presented, as quickly as possible. Typically, the speed with which subjects can perform this task is associated with the number of dots on the screen. That is, the more dots in a picture, the slower the reaction time (RT). However, when pictures are repeated, and subjects have a lot of practice at the task, eventually their RTs are no longer associated with the number of dots in a picture – subjects respond to each picture with equivalent speed (**Figure 2**). According to Logan’s (1988) theory of skill acquisition, early in training subjects count the dots and this typically takes longer to complete the more dots there are in a picture. Late in training, subjects are more likely to recognize pictures and so remember the number of dots rather than have to count them. As a result they can respond to each picture with the same speed and hence RT will not be a function of the number of dots in a picture. An RT line with a zero slope, therefore, indicates automaticity of this response.

In Lassaline and Logan’s (1993) experiments, subjects reached this state after four sessions of training (1920 trials and 64 repetitions per item). Lassaline and Logan (1993) interpreted this change in the pattern of RTs as subjects moving from a counting strategy early in practice (a controlled process) to a memory strategy later in practice (i.e., subjects recognized each picture and remembered the correct response – an automatic process).

A similar explanation invoking a transition from controlled to automatic processing has been used to account for results in the alphabet arithmetic task (Logan, 1988) and memory scanning (Schneider and Shiffrin, 1977). The fact that the pattern of performance changes that accompany practice are so easily replicated has no doubt fostered confidence in this explanation. What is not clear in any of this research, however, is the extent to which this transition occurs in individuals. The traditional approach in this area of research, as in many other areas of cognitive psychology, is to collect data from groups of subjects, and analyze the trends in the average results. Certainly, theories such as Logan’s (1988) have been developed to explain the average results. But are these theories a good explanation for what occurs in the minds of all individuals when they practice any of these tasks? It is difficult to answer this question because it is not traditional practice to publish data that reflects how well the average trends match the pattern of results produced by each individual. The aim of the experiment reported here was to provide data that could be used to answer this question with respect to the visual numerosity task.

The experiment was an attempt to replicate the results reported by Lassaline and Logan (1993) and depicted in **Figure 2**. In addition, we looked at the individual RT results of each subject to determine the extent to which the apparent transition from controlled to automatic processing occurs in a sample of people. If a result similar to that reported by Speelman and McGann (2013) was obtained – that is, that the group results replicate the classic effect but that a substantial proportion of the sample do not show the effect – then this would raise questions regarding the validity of theories designed to explain the group results.

## Materials and Methods

### Subjects

Eighteen psychology students from Edith Cowan University voluntarily participated in the study. The inclusion criteria required participants to have ‘corrected’ or ‘corrected-to-normal’ vision and English as their primary language. The participants’ ages ranged from 19 to 65 years (**Table 1**). Participants were reimbursed with a $20 shopping voucher for their time. This experiment was approved by the Edith Cowan University Human Research Ethics Committee. All subjects granted their written informed consent to participate in the experiment.

**Table 1:** Slopes of regression lines (ms/asterisk) fitted to RT data as a function of numerosity for each subject.

Participant	Age (years)	Early slope	Mid slope	Late slope	Matches auto pattern	Auto (<100 ms)
1	48	157.83 (0.42)	264.35 (0.71)	329.44 (0.66)		
2	55	221.06 (0.74)	174.39 (0.64)	147.55 (0.52)	y	
3	47	194.95 (0.21)	-21.02 (0.01)	-45.10 (0.02)	y	y
4	49	106.44 (0.23)	-16.02 (0.00)	-77.38 (0.15)	y	y
5	49	425.72 (0.94)	335.25 (0.75)	377.09 (0.80)		
6	57	247.66 (0.82)	224.68 (0.69)	263.65 (0.69)		
7	25	336.01 (0.68)	-95.78 (0.07)	-71.43 (0.07)	y	y
8	23	381.07 (0.81)	305.30 (0.81)	218.92 (0.92)	y	
9	27	264.21 (0.90)	361.59 (0.91)	250.73 (0.54)		
10	31	318.85 (0.84)	41.37 (0.09)	6.13 (0.00)	y	y
11	28	193.07 (0.38)	-15.49 (0.00)	-126.67 (0.07)	y	y
12	39	277.37 (0.85)	90.37 (0.27)	59.12 (0.08)	y	y
13	65	367.46 (0.50)	254.99 (0.32)	248.84 (0.55)	y	
14	20	309.01 (0.88)	537.37 (0.52)	232.21 (0.34)		
15	23	625.63 (0.58)	86.52 (0.13)	-54.47 (0.25)	y	y
16	19	447.66 (0.70)	551.07 (0.84)	450.31 (0.78)		
17	30	290.94 (0.83)	108.88 (0.24)	-11.66 (0.00)	y	y
18	48	216.73 (0.52)	38.69 (0.01)	-65.27 (0.12)	y	y
	
Mean/total	37.94	298.98 (0.94)	179.25 (0.57)	118.44 (0.46)	12/18	9/18
		
*r* with age		–0.42^ns^	-0.19^ns^	0.09^ns^		

*Values in parentheses are *r*^2^ values for the regression lines. Matches auto pattern: y = yes, slopes descend from Early to Mid to Late. Auto (<100 ms): y = yes, slope = 100ms or less by the Late period. ns = *p* > 0.05.*

### Design and Stimuli

The visual numerosity task used in this experiment was performed as part of a larger task used to examine transfer of training issues. Each trial had two parts. In the first part of each trial, a configuration of asterisks was presented on the computer screen. Subjects were asked to indicate the number of asterisks as quickly as possible by pressing one of six buttons on a response box. The second part of each trial involved subjects adding a number presented on the screen to the number of dots that had been presented in the previous part. Subjects then decided whether the sum was an odd or even number, indicating their decision by pressing the appropriate button on the response box. Only data from the visual numerosity part of each trial is considered in this paper.

Six stimuli were prepared for this experiment, one for each level of numerosity from 6 to 11. In each stimulus, asterisks were arranged in a pseudo-random manner, with the constraint that each asterisk was separated from other asterisks by at least 1 cm.

### Procedure

Subjects were provided with 12 trials of practice using stimuli that were not used in the experimental trials but which were similar in appearance to the experimental stimuli. Once participants fully understood the procedure the experimental trials began. In part one of each trial a fixation point appeared in the centre of the screen for 250 ms, followed by a configuration of asterisks ranging in number from 6 to 11. Subjects were required to determine the number of asterisks. The picture remained on screen until a response was made on the response box by pressing one of the keys labeled 6–11. A blank screen then followed for 250 ms. Participants were then asked to add a number to the number of stars just identified and determine whether the answer was odd or even by pressing the corresponding keys. Participants were instructed to respond as accurately and as quickly as possible. Trials were presented in blocks of six. The six trials within each block were presented in a random order. There were 50 blocks of trials, leading to 300 trials, with each stimulus being presented 50 times.

## Results

Accuracy of responses was examined to ensure that participants were not guessing with their responses. All participants maintained a mean accuracy above 80% for each block of trials.

**FIGURE 3:**
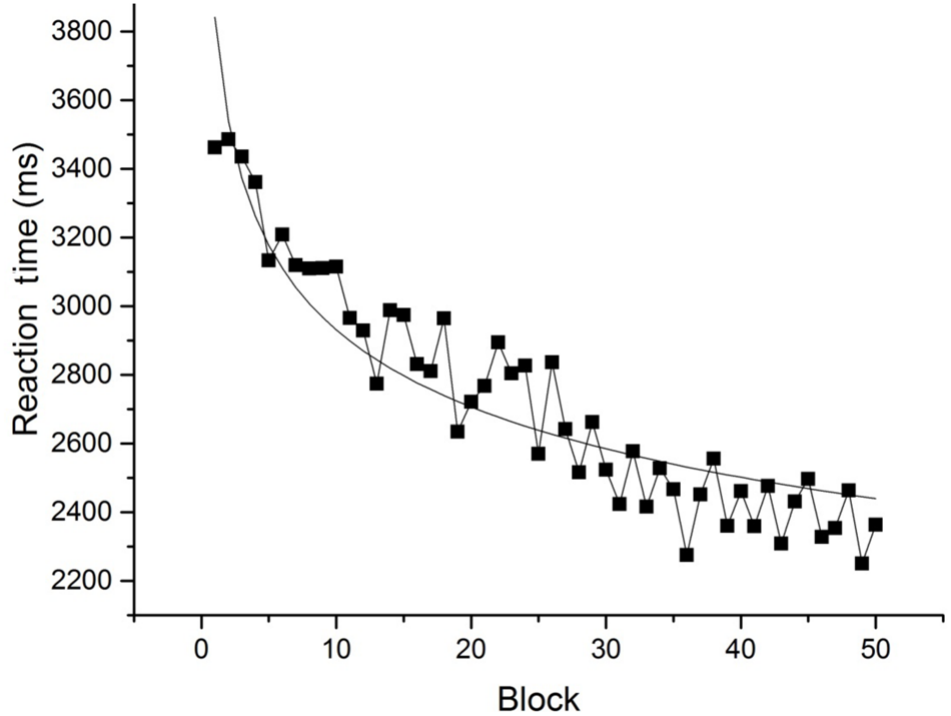
**Mean RT for each block of trials.** The smooth line is the best-fit power function (RT = 342.30 + 3500 × block^-0.13^, *r*^2^ = 0.86, and rmsd = 99.68).

Average RT across all levels of numerosity for each block was calculated. **Figure 3** shows RT as a function of practice for each block. Mean RT for the group became faster over the experiment, and is well described by a power function.

**FIGURE 4:**
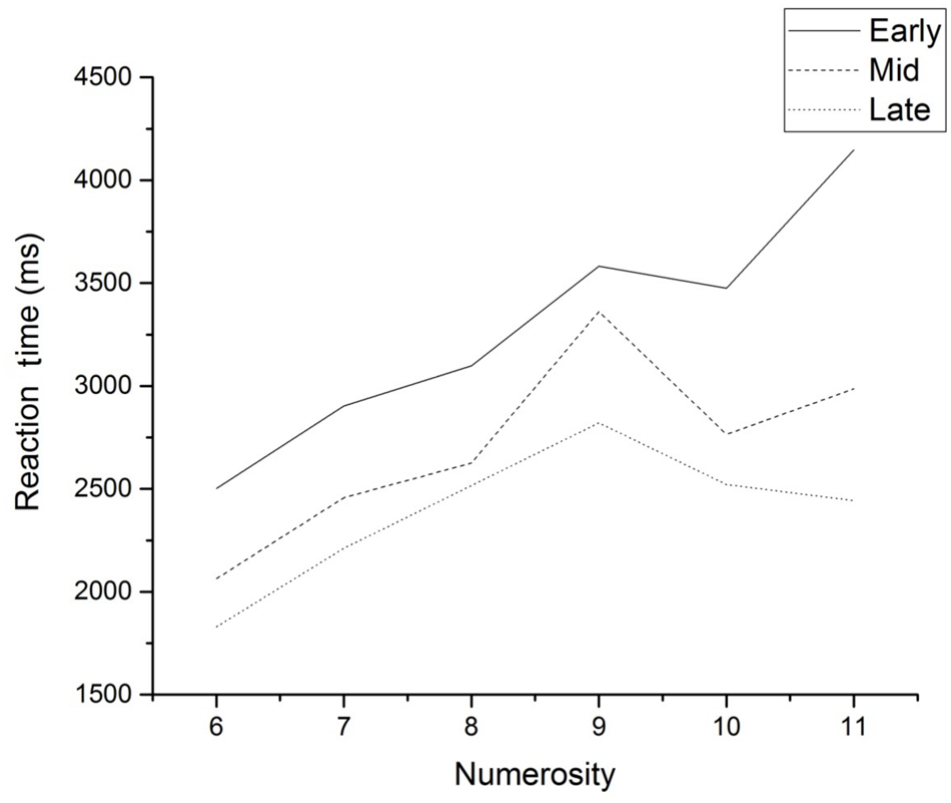
**Mean RT as a function of numerosity for the three phases of the experiment**.

Blocks were examined in phases (each block consisted of six trials): Early (blocks 1–10); Mid (blocks 21–30); and Late (blocks 41–50). Mean RT for each level of numerosity for each phase is presented in **Figure 4**. The slope of a regression line relating response latency to numerosity was calculated for each of the three phases to determine whether automaticity was reached. These values are presented in **Table 1**. Although the slope values follow the pattern of results reported by Lassaline and Logan (1993) – that is, the slopes decline in value from Early to Late in practice – the Late result does not reach 0 ms/asterisk, as would be expected if the results reflected complete automaticity. The slope value in the Late phase (118.44 ms/asterisk), however, is consistent with the slope value reported by Lassaline and Logan (1993) in several experiments after a similar amount of practice (circa 100 ms/asterisk).

Regression lines were also fitted to the RT data as a function of numerosity for each phase and for each subject. The slope values for these lines are presented in **Table 1**. Two analyses were performed with this data. The first examined the number of subjects whose slope values followed the pattern of the slopes calculated on group data. Twelve out of 18 (67%) subjects fell into this category, although subject 13 barely shows a reduction in slope value from the Mid to the Late phase. The other analysis of slope values looked at whether or not a subject reached a slope value of 100 ms/asterisk or less, to signify whether a subject had reached automaticity. This was the same cut-off point as used with the group results. The slope values of nine out of 18 (50%) subjects met this criterion.

A further test was performed to determine whether the pattern of slope value changes from phase to phase was consistent amongst the subjects. Kendall’s coefficient of concordance indicated that there was some degree of consistency amongst subjects in their slope value changes (*W* = 0.373, *X*^2^(2) = 13.444, *p* = 0.001). However, the fact that *W* was not equal to 1 indicates that there was not complete unanimity in the pattern of slope changes, and being closer to 0 than 1 supports the fact that there was a sub-set of subjects that did not show the typical pattern of slope reductions throughout training.

To explore whether some characteristic of the subjects was associated with the likelihood of them attaining automaticity, Pearson correlation coefficients were calculated between subject’s age and the regression slopes in the three practice phases. These values are reported in **Table 1**. None of these correlations were statistically significant.

## Discussion

This experiment replicated the result reported by Lassaline and Logan (1993). That is, practice with the visual numerosity task resulted in a change in the pattern of performance, with RT early in practice being a function of numerosity (i.e., the more asterisks to be counted, the longer the RT), whereas later in practice the relationship between RT and numerosity became weaker. These results can, therefore, be explained by the typical account, that performance has moved from a controlled form of processing early in practice (i.e., counting asterisks in a serial manner) to automatic processing (i.e., subjects recognize each stimulus and remember the number of asterisks in the picture). At least, this is what the group results suggest.

A different picture is apparent when the results of individual subjects are considered. First, only half of the sample produced results that suggested they had reached automaticity with the task. Second, at least one-third of the sample did not show results consistent with the group trend that replicated Lassaline and Logan’s (1993) result. Thus, for this latter sub-group, there is no evidence that their results reflected a transition from controlled to automatic processing. So, although the overall group results reflect a pattern that describes well the results of two thirds of the sample, they do not reflect the pattern of behavior in all subjects. Indeed, a sizeable minority exhibited results that suggest there was no move toward automatic performance with the visual numerosity task.

One possible explanation for why so many people in this experiment did not attain automatic performance concerns the nature of the task used in this experiment, which differed from that used by Lassaline and Logan (1993). In this experiment there were two parts to each trial. The results reported in this paper only concerned the first part of each trial, the part that matched the task used by Lassaline and Logan (1993). It is possible that the presence of the second part of each trial in this experiment may have contributed to many subjects not showing a transition to automatic performance. On the other hand, the fact that the group results for this experiment were consistent with the group results reported by Lassaline and Logan (1993) indicates that the two-part structure to each trial did not affect the overall results. It is therefore not possible to rule this explanation in or out at this stage without knowing the individual results of subjects in the Lassaline and Logan (1993) experiments. It is worth noting, though, that in other visual numerosity experiments we have conducted in our laboratory with a similar two-part structure to each trial, the group results suggested a transition from controlled to automatic processing, whereas the individual results indicated that this transition was not universal. That is, in three experiments, 7/16, 15/20, and 23/40 people showed a transition to automaticity.

Another possible point of difference between our experiment and those reported by Lassaline and Logan (1993) concerns age. Lassaline and Logan (1993) did not report the ages of their subjects, only that they were undergraduate Psychology students. Our subjects also were undergraduate Psychology students, however, given the age profile of students at Edith Cowan University, the age range of our subjects being 19–65 years is not unusual. It may well be the case that the age range of our subjects is larger than the age range of subjects in Lassaline and Logan’s (1993) experiments; however, age was not correlated with our measure of automaticity (regression line slopes) at any point in the experiment, and so cannot explain why so many subjects did not attain automaticity.

At the least, this experiment questions the conclusions that can be drawn from group results on the visual numerosity task. Although the group results are consistent with a transition from controlled to automatic processing, they do not reflect the performance of all subjects in the group. Even though there was a similar number of repetitions per item in this experiment (50 repetitions/item) to that in Lassaline and Logan’s (1993) experiments (up to 64 repetitions/item), and so a similar opportunity to attain automaticity, some subjects in this experiment showed no evidence of moving toward automatic processing. Indeed, it seems that these people continued to count asterisks throughout the experiment. Thus, the transition from controlled to automatic processing as a result of practice with a task, which is a feature of many theories of skill acquisition (e.g., Logan, 1988; Anderson and Lebiere, 1998), may not be an automatic feature of skill acquisition, at least for some people. Other work has demonstrated that not all experimental subjects adopt more efficient performance strategies when acquiring skills, but rather just improve the application of a less-efficient strategy (Rowell et al., 2015). It is now an open question as to why some people do not exhibit this transition when many others do. Importantly, this is a question that would never arise without attention to differences between individual and group results (Speelman and McGann, 2013).

## Conflict of Interest Statement

The authors declare that the research was conducted in the absence of any commercial or financial relationships that could be construed as a potential conflict of interest.
